# A standardised experimental setup for simulating ocean warming and acidification in benthic marine invertebrates

**DOI:** 10.3897/BDJ.14.e185047

**Published:** 2026-02-09

**Authors:** Eva Chatzinikolaou, Panagiotis Grigoriou, Athanasios Anastasiadis, Emmanouela Vernadou, Thanos Dailianis

**Affiliations:** 1 Hellenic Centre for Marine Research (HCMR), Institute of Marine Biology, Biotechnology and Aquaculture (IMBBC), Heraklion, Crete, Greece Hellenic Centre for Marine Research (HCMR), Institute of Marine Biology, Biotechnology and Aquaculture (IMBBC) Heraklion, Crete Greece; 2 Hellenic Centre for Marine Research (HCMR), Cretaquarium, Heraklion, Crete, Greece Hellenic Centre for Marine Research (HCMR), Cretaquarium Heraklion, Crete Greece

**Keywords:** experimental set-up, laboratory monitoring conditions, climate change, common garden experiment, sessile invertebrates

## Abstract

Recent studies identify ocean warming and acidification as major drivers of ecological change in the Eastern Mediterranean, posing serious threats to marine biodiversity, particularly for sessile or low-mobility organisms that cannot escape unfavourable conditions. At the same time, the need for standardised experimental approaches capable of generating high-quality data on organismal responses to multiple climate stressors has become increasingly evident. This manuscript presents a fully detailed and replicable experimental framework for simulating ocean warming and acidification in benthic marine invertebrates under controlled laboratory conditions. Detailed protocols include the technical set-up, experimental design, selection of climate scenarios, monitoring procedures and criteria for species selection and demonstrating its application through a validation case study from the MACCIMO project.

## Introduction

Climate change is increasingly altering the physical and chemical conditions of the world’s oceans, with significant consequences for marine organisms. Rising sea temperatures disrupt metabolic rates, reproductive cycles and migration patterns, forcing many species to shift their distributions into deeper and cooler waters (Poloczanska et al. 2013). Ocean acidification, driven by elevated CO₂ absorption, decreases carbonate ion availability, impairing calcification in corals, molluscs and planktonic organisms (Doney et al. 2009). These interacting stressors collectively threaten marine biodiversity and the overall stability of ocean ecosystems (Pörtner et al. 2014). Sessile organisms or organisms with restricted mobility, such as sponges and benthic gastropods, are more vulnerable to the impacts of climate change, since they rely on local conditions and cannot relocate to avoid unfavourable or extreme conditions. Sessile marine organisms are usually characterised by a short mobile phase during their larval stage and they have limited options for habitat choice, thus they are often incapable of undergoing physiological or morphological adaptation to acclimatise to varying environmental conditions (Palumbi 1986).

Intra-specific diversity, especially evolutionary traits fixed within sub-groups of the same species, can alter tolerance levels in populations exposed to varying environmental regimes, thus promoting resilience to local conditions (Palumbi et al. 2014). This is especially relevant in the hydrologically complex Mediterranean system, where environmental gradients are intrinsically strong and are expected to become even more variable in the near future (Giorgi and Lionello 2008, Denaxa et al. 2025). Common garden experiments involve rearing individuals of the same species from different locations under identical experimental conditions, allowing observed differences in morphological, physiological or other traits to be attributed primarily to genetic differences (nature) or environmental history (nurture) (de Villemereuil et al. 2015, Rivi et al. 2022). This experimental approach facilitates control of local adaptation, phenotypic plasticity and evolutionary potential in order to study the genetic bases of complex traits (e.g. life history, morphological and physiological traits) (de Villemereuil et al. 2015).

Recent regional syntheses indicate that ocean warming and acidification are key drivers of ecological change in the Eastern Mediterranean, with wide-ranging consequences for marine biodiversity (Christou et al. 2025). However, there is a critical need for standardised experimental frameworks that can produce high-resolution, mechanistic data on organismal responses to combined climate stressors, in order to move beyond observational and model-based studies and provide better information for biodiversity forecasts and conservation strategies. Here, we present a fully detailed, replicable experimental platform for simulating climate change stressors (i.e. warming and acidification) in sessile or semi-motile benthic marine invertebrates. This experimental platform has been used to generate foundational datasets for subsequent morphology, physiological, gene expression and symbiosis studies and can be adapted broadly for future investigations where quantitative experimental data are urgently needed. Besides the technical set-up, the experimental approach used to perfom in situ laboratory experiments for climate change, is also important for the success of these studies. Other steps that need to be considered for the replication of such experiments are the selection of climate change scenarios, the verification of their appropriate monitoring and the selection of suitable experimental organisms.

The present manuscript describes the experimental protocols, equipment set-up and scenarios used in the climate change studies performed within the project MACCIMO (Multi-level Approaches to assess Climate Change Impact to Marine Organisms). Specific details on selected temperature and pH conditions, experimental invertebrate species, locations and samples collection, are presented as validation examples of the generalised protocol. This manuscript will provide the methodological basis for all the subsequent publications arising from the MACCIMO project that will be included in the special issue "Multi-level assessment of climate change impacts in benthic marine invertebrates: insights from the MACCIMO project". In addition, it can be used as a standardised experimental framework that can be easily replicated in other climate change simulation studies.

## Implementation and Methodology

### Step 1. Selection of experimental conditions – Climate Change scenario

The selection of Climate Change scenarios is crucial for the simulation of future projected environmental conditions under in-situ laboratory experiments. It is important to select realistic and well justified temperature and pH conditions, to foresee for an acclimatisation period in order to avoid misleading stress to the organisms, as well as to establish a standardised control treatment to support comparisons. The scenario of the present study was based on the "high GHG emissions" RCP 8.5 scenario of IPCC as this is described in the Climate Change 2023: Synthesis Report (Calvin et al. 2023). Temperature was increased by 4°C and pH was decreased by 0.3 units. According to the IPCC Report, these conditions will be a fact in eastern Mediterranean by the end of the 21^st^ century if no effort is made to reduce CO_2_ emissions in the coming years.

**Figure 1.**
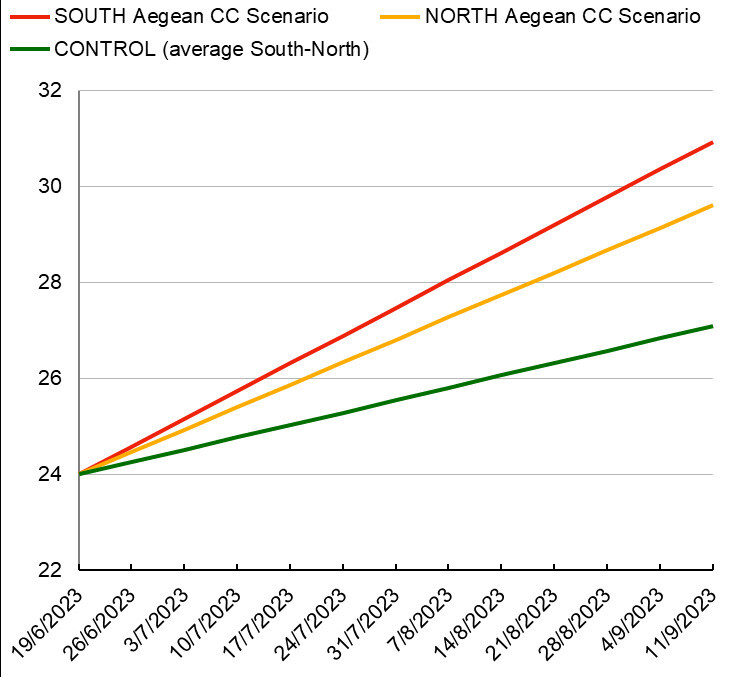
Temperature scheme for the three experimental treatments, combining increased temperatures with low pH for Climate Change (CC) scenarios.

More specifically, three climate change scenarios were simulated during the experiments (Fig. 1): 1) the Control scenario in which the ambient temperature was estimated as the average of the summer temperatures (27°C) between the two populations (North and South Aegean). The pH was ambient (~ 8.1); 2) the South Aegean Climate Change (SACC) scenario ("extreme") in which temperature was estimated as the maximum recorded in South Aegean (Crete) during summer (27°C) increased by 4°C. The pH was decreased by 0.3 units (~ 7.8); and 3) the North Aegean Climate Change (NACC) scenario ("mild") in which temperature was estimated as the maximum recorded in North Aegean (Chalkidiki) during summer (26°C) increased by 4°C. The pH was decreased by 0.3 units (~ 7.8).

**Figure 2.**
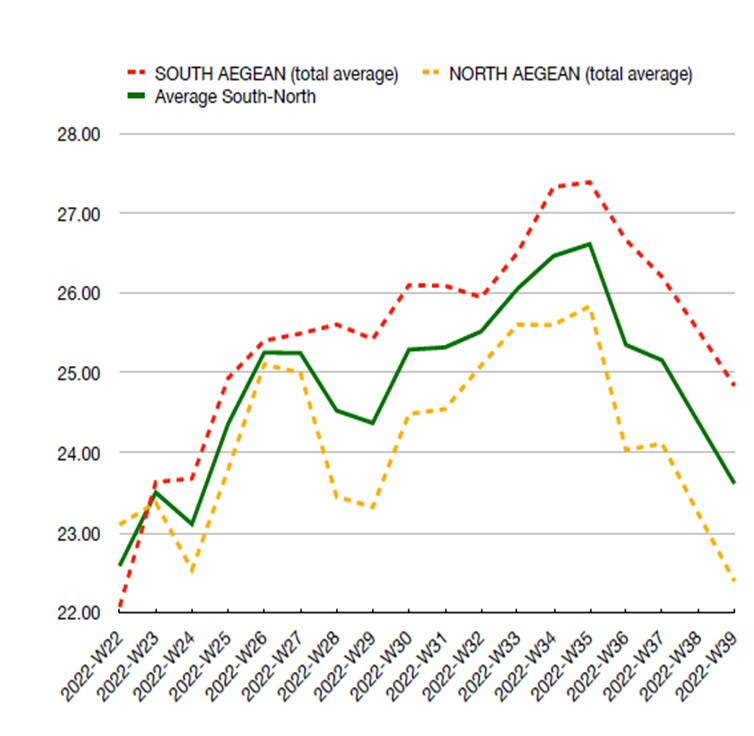
Seawater temperatures in the North and South Aegean Sea during summer (data by Poseidon System for year 2022).

**Figure 3.**
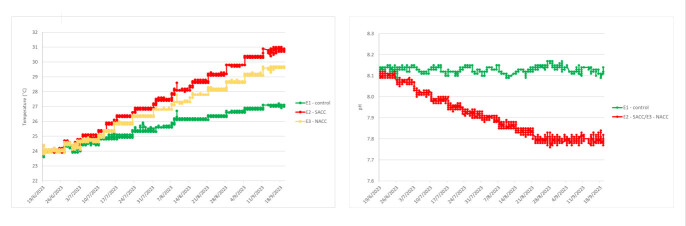
Experimental conditions for the Control scenario, the South Aegean Climate Change (SACC) scenario and the North Aegean Climate Change (NACC) scenario, regarding temperature (A) and pH (B).

All scenarios, regarding the starting temperature points, were based on the true values of temperatures recorded in the respective areas during the summer of year 2022 (data source: Poseidon System, HCMR) (Fig. 2). Temperature during the experiment was following the ambient rate of increase during the summer months, estimated at 0.26°C/week for the control, 0.58°C/week for SACC and 0.47°C/week for NACC. Starting temperature in all treatments was 24°C, which was close to the average ambient temperature during the start of the experiment in order to avoid acclimatisation stress. The final temperature for the control treatment was 27°C, for the SACC, it reached 31°C and for NACC, it was 30°C (Fig. 3A). The total duration of the experiment was three months.

The pH in the two Climate Change treatments (SACC and NACC) was decreased by a rate of 0.03 units/week in order to avoid acclimatisation stress (Fig. 3B). The starting pH in all treatments was ~ 8.1. The final pH for the Control remained constant at around 8.1, while, for the Climate Change treatments (SACC and NACC), it gradually dropped to 7.8.

### Step 2. Experimental set-up and equipment

**Figure 4.**
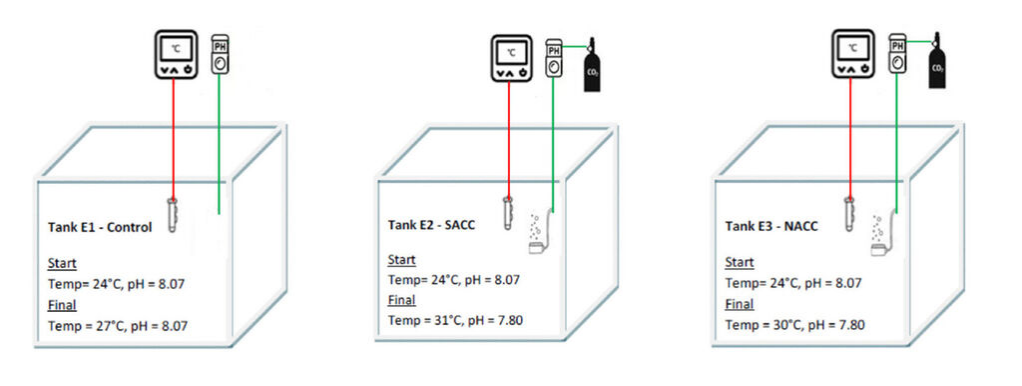
Set-up of the three experimental tanks and adjusted experimental conditions.

A semi-closed experimental system (Fig. 4) installed in the Hellenic Centre for Marine Research was used to ensure controlled experimental conditions. Temperature and pH were continuously monitored, recorded and controlled by a GHL Profilux 4 controller system. The deviation of the set temperature values was ± 0.3°C and ± 0.01 for the pH (controller’s capacity).

Temperature and pH sensors were connected to the three experimental tanks (E1, E2, E3). Submerged aquarium heaters (Eheim 200W) were used to raise the temperature according to the desired level in the treatments. A bubbling CO_2_ system attached to CO_2_ bottles (40 litres) through a manometer (Mufan) and connected to the controller system (GHL Profilux 4) was used to adjust the low pH treatments (tanks E2 and E3). The tanks were covered with plastic lids in order to minimise gas exchanges that could alter *P*CO_2_ and affect the controlling of the desired pH conditions. The pH electrodes were calibrated once a week with 7.00 and 9.00 GHL buffers.

A wave maker pump (Sicce Voyager NANO), an air lift system and additional air stones have been used to ensure a sufficient level of water circulation. The water in the experimental tanks was renewed by 50% twice a week, thus maintaining a high water quality within the system (NO_2_ < 0.1 mg/l, NO_3_ < 0.5 mg/l, NH_3_/NH_4_ < 0.2 mg/l). Nitrogenous waste products were also assessed twice a week by using a Hanna HI83303 Multiparameter photometer. Besides the controlled temperature and pH conditions, all other parameters remained constant in order to avoid any interference with the experiment. Salinity was maintained at 40.0 ppt, oxygen at 7 mg/l, the photoperiod was 12 h light/12 h dark and light intensity was 50 lux. Temperature and pH (on the National Bureau of Standards scale - pHNBS) were additionally measured daily (3420 WTW multi-meter), while salinity (salinometer) and oxygen (OxyGuard) were checked every two days. Total alkalinity (TA) was measured every two weeks according to the Standard Operating Procedure (SOP 3b) as described in Dickson et al. (2007) using an open-cell titration (Metrohm Dosimat 765; 0.1 mol/kg in HCl).

### Step 3. Selection of species

**Figure 5.**
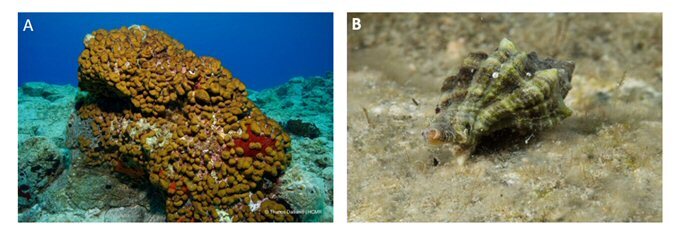
The experimental organisms, the sponge *Chondrilla nucula* (A) and the gastropod *Hexaplex trunculus* (B). Photos by T. Dailianis.

The experimental set-up described in this manuscript has been designed to host small marine invertebrates, especially sessile or semi-motile ones. Experimental organisms should be selected according to several criteria, such as availability of local populations, easily maintained under laboratory conditions and significant environmental or commercial value. In the present case study, the sponge *Chondrilla nucula* Schmidt, 1862 and the gastropod *Hexaplex trunculus* (Linnaeus, 1758) were used in the experiments performed (Fig. 5).

*Chondrilla nucula* is a photophilic sponge (Milanese et al. 2003) and a widespread species found across a variety of habitats usually in coastal waters (Strano et al. 2020). This sponge plays an important role in maintaining water quality (Milanese et al. 2003) and is also of great commercial interest for biotechnological applications (Chelossi et al. 2007). *Hexaplex trunculus* is a cosmopolitan species found in the intertidal and subtidal zone and it is well adapted and resistant to changing environmental conditions (Lahbib et al. 2010). It is a commercial edible species (Vasconcelos et al. 2006, Lahbib et al. 2012) and an ecological indicator for organotin compounds pollution (Abidli et al. 2012). Owing to their shallow distribution, both species are potentially exposed to temperature spikes, marine heatwaves and progressive increases in sea-surface temperature, making them suitable model organisms for climate-stress experiments.

The protocol described here can be readily applied to a broad range of small sessile or semi-motile marine invertebrates beyond sponges and gastropods, including bryozoans, cnidarians, echinoderms and polychaetes. Such organisms are often vulnerable to elevated summer temperatures and extreme heat events and experimental assessment of their responses to combined climate stressors is essential for improving our understanding of future marine ecosystem trajectories.

### Step 4. Field collection and maintenance of experimental organisms

Additional activities that need to be planned carefully include field sampling, transport of live organisms in the laboratory, accommodation in appropriate containers and random separation of samples.

Individuals of both species were collected from two locations in the South Aegean (Crete, 35.3357° N, 25.2815° S, up to 5 m depth) and the North Aegean (Chalkidiki, 39.9315° N, 23.7348° S, up to 5 m depth), by means of scuba or free diving. Although *C. nucula* can proliferate asexually through fragmentation, enabling it to cover extended areas over a single rocky surface, it also reproduces sexually via internal fertilisation and the release of embryos into the seawater (Sidri et al. 2005). Sponge larvae can subsequently crawl over the substrate or be transported by wave action and currents to new settlement surfaces (Mariani et al. 2006). To ensure that collected sponge fragments originated from different individuals, *C. nucula* patches separated by a distance greater than 5m were selected. Collected specimens were transported live to the experimental facilities, in individual aerated bags placed in cooler boxes.

**Figure 6.**
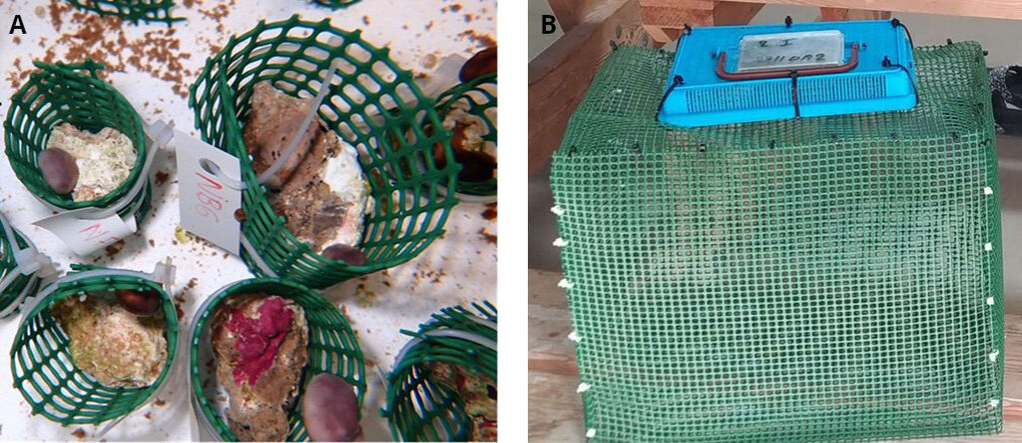
Maintenance of sponges (A) and gastropods (B) in the experimental facilities. Photos by E. Vernadou and A. Anastasiadis.

**Figure 7.**
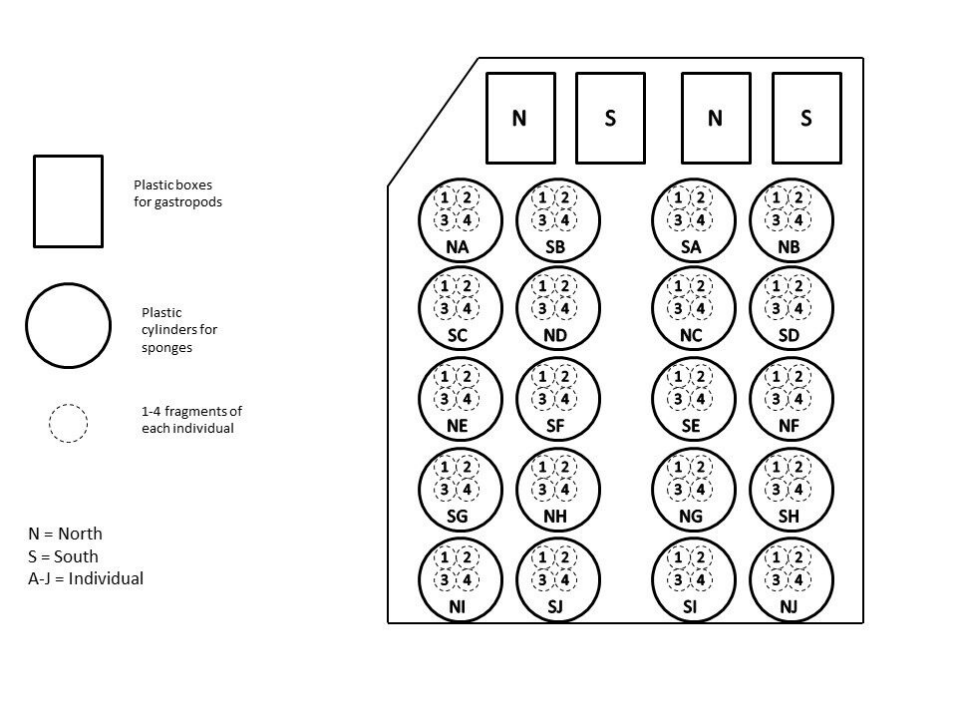
Set-up of each experimental tank regarding the positioning of samples.

A total of 40 fragments of *C. nucula* (originating from 10 individual patches) and 40 individuals of *H. trunculus* from each of the two populations (South and North) were placed in each experimental-treatment tank (150 litres) in order to ensure the collection of samples for all the required analyses and measurements and also to cover possible losses due to unforeseen mortalities. The gastropods were placed inside rectangular boxes made from plastic net, while the fragments of sponges were placed inside cylindrical plastic nets on which they were able to attach (Fig. 6). The sponges were randomly separated in groups as shown in Fig. 7, where each cylindrical net contained four sponge fragments of the same individual. A stone was placed on the bottom of each net serving as additional settlement substrate for the organisms and also keeping the small cages submerged.

The organisms must be maintained under optimum well-being conditions during the experiment. Gastropods were fed ad libitum three times per week with freshly opened mussels and the sponges were fed two times per week with a solution of *Chlorella* sp. (40 x 10^6^ cells/ml).

### Step 5. Acquisition of samples for downstream analyses

The acquisition of samples to support downstream analyses (assessment of morphological traits, metabolic rate, microsymbiont communities and gene expression) was performed following the end of the experiment after three months. Samples for respiration experiments (metabolic rate) were used alive and introduced in metabolic chambers. For all other instances, specimens were anaesthetised using a rising concentration of magnesium chloride (MgCl_2_) starting at 1.5% and gradually reaching 3.5% according to the European Directive 2010/63 EU on the protection of animals used for scientific purposes. The samples can then be stored frozen until further anaysis is performed. In the present study, samples were stored at -20 ͦC for micro-CT scanning and DNA extraction or were snap-frozen in liquid nitrogen and stored at -80°C for RNA extraction.

## Conclusions

The experimental set-up, protocols and climate scenarios presented in the current manuscript support the implementation of controlled laboratory experiments for studying the impact of ocean warming and acidification in small sessile and semi-motile marine sessile invertebrates. A wide range of studies - from morphological to physiological and from gene expression to the assessment of microsymbionts - were developed following this experimental approach, which can be easily replicated under different climate scenarios and experimental species. A variety of marine taxa, such as sponges, cnidarians, polychaetes, echinoderms, molluscs and bryozoans can be used in similar experiments which can run either in the short (e.g. simulating heatwaves) or in the long term (seasonal simulation). The experimental system is semi-enclosed and easily sustainable and can accommodate a sufficient number of biological replicates depending on the tank size. The controller system offers efficient monitoring of the parameters (temperature and pH) which can be well adjusted to the desired levels with high accuracy. The described protocols provide a practical and replicable foundation for future experimental studies investigating organismal responses to climate-related stressors.

This is the first manuscript in a series of publications under the special issue "Multi-level assessment of climate change impacts in benthic marine invertebrates: insights from the MACCIMO project". The protocols presented in this manuscript provide a detailed description of the general methodological and equipment set-up which supported the experiments performed and provided samples to all the associated downstream studies of the project MACCIMO.

## Project description

**Project title**: Multi-level Approaches to assess Climate Change Impact to Marine Organisms (MACCIMO)

**Project aim**: The experiments performed during the project MACCIMO followed an integrative and multi-level approach aiming to study the impact of ocean warming and ocean acidification on different marine invertebrate taxa with partial or low motility, namely the sponge *Chondrilla nucula* (Porifera, Demospongiae) and the gastropod *Hexaplex trunculus* (Mollusca, Gastropoda). The different mechanisms - molecular and physiological - that are activated for organisms to cope with the imposed stress factors were examined, in combination with alterations in structural morphology and associated microsymbiotic communities. A common garden experiment was conducted to assess the responses of these organisms to thermal and oxidative stress, with the additional aim of investigating intraspecific variation in these responses and thereby uncovering potential adaptive diversity amongst geographically distinct populations. The hypothesis examined was that the climate change treatments are expected to induce more pronounced changes to individuals originating from northern Aegean locations, where the effects of climate change are currently less pronounced (Skliris et al. 2011), while the southern populations would expectedly be more resilient to cope with extreme environmental conditions.

**Study area description**: Crete (South Aegean), Chalkidiki (North Aegean), Mediterranean Sea, Greece

**Personnel**: Dr Thanos Dailianis (scientific responsible, experimental design, sampling, manuscript review), Dr Eva Chatzinikolaou (WP2 leader, experimental design, manuscript writing), Dr Panagiotis Grigoriou (experimental design, manuscript review), Emanouela Vernadou (sampling, technical support), Athanasios Anastasiadis (sampling, technical support)

**Taxonomic coverage**:

Phylum: Mollusca, Class: Gastropoda, Order: Neogastropoda, Family: Muricidae, Genus: *Hexaplex*, Species: *Hexaplex trunculus*

Phylum: Porifera, Class: Demospongiae, Order: Chondrillida, Family: Chondrillidae, Genus: *Chondrilla*, Species: *Chondrilla nucula*
